# Diffuse small bowel thickening in aids patient - a case report

**DOI:** 10.1186/1471-2334-10-310

**Published:** 2010-10-28

**Authors:** Rohit Singla, Samriti Hari, Surendra K Sharma

**Affiliations:** 1 Department of Medicine, All India Institute of Medical Sciences, New Delhi, India; 2 Department of Radio Diagnosis, All India Institute of Medical Sciences, New Delhi, India, Study conducted at All India Institute of Medical Sciences, New Delhi-110029, India

## Abstract

**Background:**

Diarrhea is common in HIV/AIDS patients, caused by both classic enteric pathogens and different opportunistic agents. *Infection with these different pathogens may lead to similar radiological findings, thus causing diagnostic confusion*.

**Case presentation:**

A 30-yr-old female with AIDS presented with chronic diarrhea of 4 months duration. She had diffuse small bowel thickening present on CT scan of her abdomen, with stool examination showing no parasites. She was erroneously diagnosed as abdominal tuberculosis and given antituberculosis drugs with which she showed no improvement. Repeat stool examination later at a specialized laboratory revealed *Cryptosporidium parvum *infection.

The patient was given an extended course of nitazoxanide treatment, as her stool examination was positive for *Cryptosporidium parvum *even after 2 weeks of drug consumption. Parasite clearance was documented after 10 weeks of treatment. Interestingly, the bowel thickening reversed with parasitological clearance.

**Conclusions:**

*Cryptosporidium parvum *may lead to small bowel thickening in AIDS patients. This small bowel thickening may reverse following parasitological clearance.

## Background

Diarrhea is a common manifestation of AIDS [1,2] (acquired immunodeficiency syndrome), causing significant morbidity and mortality. Diarrhea in AIDS patients is caused by both classic enteric pathogens and different opportunistic agents. *Infection with these different pathogens may lead to similar radiological findings, thus causing diagnostic confusion*.

*Cryptosporidium parvum *is a common cause of diarrhea in AIDS patients [3]. The fact that it may cause diffuse small bowel thickening has been reported by Redvanly *et al *[4], but is not widely recognized. Limited treatment options are available against this parasite. Nitazoxanide is the only drug approved for treatment of this disease. However, there is not much data supporting its efficacy in immunocompromised individuals. Also, there are no clear guidelines stating the exact duration of treatment with this drug.

## Case presentation

A 30-yr-old female, diagnosed case of AIDS, was admitted for evaluation of weight loss and intractable diarrhea of 4 months duration. The patient complained of chronic, painless, nonbloody, profuse watery diarrhea with a weight loss of 10 kg *(decrease to 30 kg from 40 kg prior to illness)*. She had no complaints of fever, cough or expectoration.

**Figure 1:**
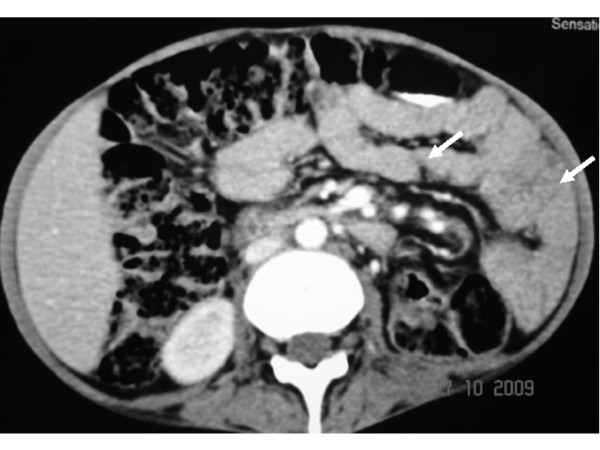
**Axial contrast enhanced CT image shows diffuse, concentric wall thickening of multiple small bowel loops (arrows)**.

Previously, during evaluation of her complaints in the outpatient department, she was discovered to be HIV (human immunodeficiency virus) infected with a CD4 count of 38 cells/μl. She also had anemia with hemoglobin value of 8 g/dl, normal kidney function, normal AST/ALT (aspartate aminotransferase/alanine aminotransferase) and elevated serum alkaline phosphatase. Stool examination showed no red blood cells or leukocytes, and no ova or cysts were observed. A contrast enhanced CT scan of the abdomen (figure 1) showed diffuse small bowel wall thickening, a fatty liver and no significant lymphadenopathy or ascites. Her chest radiograph was normal. Based on these details, a diagnosis of abdominal tuberculosis was made 2 months back, and she was started on standard 4-drug regimen for treatment of tuberculosis. Besides this, she was also given co-trimoxazole and once a week azithromycin prophylaxis for *Pneumocystis jiroveci *and *mycobacterium avium-intracellulare *infection respectively. Two weeks later she was started on non-zidovudine based HAART (highly active antiretroviral therapy), after she had tolerated antituberculosis drugs. However diarrhea failed to improve, and she continued to lose weight.

During this admission her stool sample was sent to a specialized microbiology laboratory, where acid fast oocysts of *Cryptosporidium parvum *were observed. Her antituberculosis drugs were stopped and she was started on treatment with tablet Nitazoxanide 500 mg twice a day. Her peripheral smear showed a megaloblatic anemia (mean corpuscular volume - 120 fl), for which folate and vitamin B_12 _were supplemented. Her kidney functions, AST/ALT were again normal, but she had raised serum alkaline phosphatase and a low serum albumin of 2.8 g/dl. In hospital she was also given supportive care with oral rehydration solution, antidiarrhoeals, and a nutritious diet low in lactose. After 2 weeks of nitazoxanide treatment the patient's stool remained positive for cryptosporidium. Her hemoglobin level had improved with folate and vitamin B_12 _supplementation. At this moment it was decided to discharge her on extended nitazoxanide treatment while continuing HAART, antibiotic prophylaxis and folate and vitamin B_12_, with monthly follow up.

**Figure 2:**
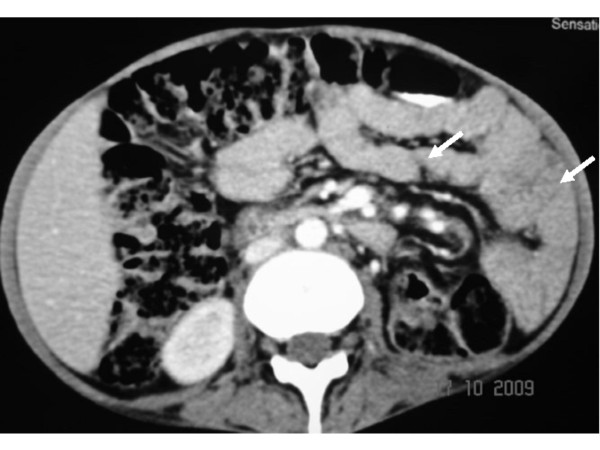
**Axial contrast enhanced CT image shows normal small bowel loops after 10 weeks of antiparasitic therapy (arrows)**.

Patient was again observed 2 months after discharge. Her diarrhea had settled for last 1 week. Her stool this time was negative for *Cryptosporidium parvum*, which was confirmed twice. Repeat CT scan of the abdomen (figure 2) showed resolution of the small bowel thickening, persistent fatty liver, no lymphadenopathy or ascites. A repeat CD4 count was done which was 36 cells/μl only.

Following parasitological clearance, the patient showed improvement in appetite and gained 5 kg weight over next one month. HAART and vitamin supplementation was continued. In view of lack of definite guidelines on secondary prophylaxis, the authors decided to continue nitazoxanide until the patient would achieve a CD4 count of 100 cells/μl.

## Discussion

The number of HIV infected individuals is continuing to increase worldwide. World Health Organization estimated that 33 million individuals worldwide were HIV infected at the end of year 2007 [5]. Diarrhea is a common manifestation of this disease, causing significant morbidity and mortality [2,3]. Superimposed infection by both classic pathogens and different opportunistic agents results due to defective immunity, leading to diarrhea.

*Cryptosporidium *is a protozoan parasite which causes a self-limited diarrhoeal illness in immunocompetent individuals and chronic, intractable diarrhea in AIDS patients; especially those with a CD4 count < 100/ul. Typically, diarrhea is profuse, watery, nonbloody, leading to fluid and electrolyte depletion. Stool examination shows no red blood cells or leukocytes. In a recently done study at Pune in India [6], 16 (12%) of 137 consecutive HIV infected patients with diarrhea had *Cryptosporidium parvum *infection.

Cryptosporidial infection has a predilection for the proximal small bowel, resulting in nonspecific thickening of the duodenum, jejenum and proximal ileum [4]. Multiple loops of fluid-filled and thickened small bowel loop can be identified on CT. Lymphadenopathy is not a feature of the disease [4]. Infection with *Isospora belli *can result in a gastrointestinal disease that is clinically and radiologically indistinguishable from cryptosporidiosis [7]. Small bowel thickening in AIDS patients may also be seen due to small bowel lymphoma [8] or infection with *mycobacterium avium-intracellulare *[9,10], both of which are associated with lymphadenopathy. *Mycobacterium tuberculosis *infection results in necrotic lymph nodes on CT in 90% of patients, with focal hepatic and splenic lesions, ascites and distal ileal thickening [9]. Hence, the finding of diffuse small bowel wall thickening with no lymphadenopathy and no focal lesions in liver/spleen in our case argued against a diagnosis of tuberculosis.

Our case also shows that the small bowel thickening seen on CT abdomen resolved with parasitological clearance. Such a result is expected, but not previously reported in English literature.

Limited treatment options are available which have shown success in eradicating this parasite in immunocompromised patients. In a double-blind randomised controlled trial in Mexico [11], nitazoxanide 1-2 g daily for 2 weeks resulted in parasitologic cure in 65% of treated patients. Another trial done at United States [12] supported the use of nitazoxanide in AIDS patients with diarrhoea. However, a recently conducted Cochrane review [13] which included 7 trials involving 169 patients showed no reduction in the duration or frequency of diarrhoea by nitazoxanide compared with placebo. Also, there was a lot of heterogeneity in drug dosage and duration of treatment. *The CDC guidelines *[14]*presently recommend nitazoxanide along with antiretroviral treatment for AIDS patients with cryptosporidiosis. These antiparasitic drugs, however, should never be used as a substitute for HAART in the treatment of AIDS-associated cryptosporidiosis*.

Our case shows that extended treatment with nitazoxanide may lead to parasitological clearance, even in face of low CD4 count. *In our case, the lack of rise in CD4 count, despite consuming 4 months of HAART can be explained by various factors. It could be ascribed to malabsorption of drugs due to chronic diarrhea. Additionally, non-adherence may also explain poor response to HAART in addition to malabsorption. Lack of rise in CD4 count supports the notion that parasitological clearance was likely because of nitazoxanide, and not secondary to immune reconstitution. However, change in CD4 count is an indirect measure of efficacy of HAART. This observation in the absence of demonstration of concomitant rise in viral load does not definitively prove that HAART was not efficacious*.

Paromomycin and azithromycin have also been tried, with little data supporting their efficacy. Best treatment option remains immune reconstitution with HAART. This results in excellent clinical responses as assessed by stool frequency, weight gain, and clearance of oocysts from the stool. However, rapid relapse after discontinuation of antiretroviral therapy suggests that cryptosporidial infection is suppressed rather than cured [15,16]. Supportive care with antidiarrhoeals, correction of fluid and electrolyte disturbance, and a nutritious diet low in lactose should also be provided to these patients.

## Conclusions

*Cryptosporidium parvum *is a frequent cause of diarrhea in AIDS patients, especially those with a CD4 count < 100/ul. It may lead to small bowel thickening, which should not be ascribed to other etiologies in an appropriate clinical setting. This small bowel thickening may reverse following parasitological clearance. Limited treatment options are available against this pathogen. Extended treatment with nitazoxanide *along with HAART *may help in achieving parasitological clearance.

## Abbreviations

AIDS: acquired immunodeficiency syndrome; ALT: alanine aminotransferase; AST: aspartate aminotransferase; HIV: human immunodeficiency virus;

## Consent

Written informed consent was obtained from the patient.

## Competing interests

The authors declare that they have no competing interests.

## Authors' contributions

RS was involved in patient care, and was a major contributor in writing the manuscript. SH was the radiologist who interpreted the CT films. SKS was involved in writing the manuscript and patient care. All authors have read and approved the final manuscript

## Pre-publication history

The pre-publication history for this paper can be accessed here:

http://www.biomedcentral.com/1471-2334/10/310/prepub

## References

[B1] Janoff EN Smith PD Prospectives on gastrointestinal infections in AIDS Gastroenterol Clin North Am 1988 17 451 63 3049355

[B2] Framm SR Soave R Agents of diarrhea Med Clin North Am 1997 81 427 47 10.1016/S0025-7125(05)70525-3 9093236

[B3] Wiwanitkit V Intestinal parasitic infections in Thai HIVinfected patients with different immunity status BMC Gastroenterol 2001 1 3 5 10.1186/1471-230X-1-3 11394966 PMC32247

[B4] Redvanly RD Silverstein JE Intra-abdominal manifestations of AIDS Radiol Clin North Am 1997 35 1083 125 9298089

[B5] UNAIDS 2008 report on the global AIDS epidemic 2008 Geneva; UNAIDS

[B6] Kulkarni SV Kairon R Sane SS Padmawar PS Kale VA Thakar MR Mehendale SM Risbud AR Opportunistic parasitic infections in HIV/AIDS patients presenting with diarrhoea by the level of immunesuppression Indian J Med Res 2009 130 63 66 19700803

[B7] Goodgame RW Understanding intestinal spore forming protozoa: cryptosporidia, microsporidia,isospora and cyclospora Ann Intern Med 1996 124 429 41 8554253 10.7326/0003-4819-124-4-199602150-00008

[B8] Radin DR Esplin JA Levine AM Ralls PW AIDS related non-Hodgkin's lymphoma: abdominal CT findings in 112 patients AJR 1993 160 1122 39 10.2214/ajr.160.5.8470595 8470595

[B9] Radin DR Intraabdominal Mycobacterium tuberculosis vs Mycobacterium avium-intracellulare infections in patients with AIDS: distinction based on CT findings AJR 1991 156 487 91 1899742 10.2214/ajr.156.3.1899742

[B10] Nyberg DA Federle MP Jeffrey RB Bottles K Wofsy CB Abdominal CT findings of disseminated Mycobacterium avium-intracellulare in AIDS AJR 1985 145 297 9 3875228 10.2214/ajr.145.2.297

[B11] Rossignol JF Hidalgo H Feregrino M Higuera F Gomez WH Romero JL Padierna J Geyne A Ayers MS A double-blind placebo-controlled study of nitazoxanide in the treatment of cryptosporidial diarrhoea in AIDS patients in Mexico Trans R SocTrop Med Hyg 1998 92 663 66 10.1016/S0035-9203(98)90804-5 10326116

[B12] Rossignol JF Nitazoxanide in the treatment of acquired immune deficiency syndrome-related cryptosporidiosis: results of the United States compassionate use program in 365 patients Aliment Pharmacol Ther 2006 24 887 94 10.1111/j.1365-2036.2006.03033.x 16918894

[B13] Abubakar II Aliyu SH Arumugam C Hunter P Usman N Prevention and treatment of cryptosporidiosis in immunocompromised patients Cochrane Database of Systematic Reviews 2007 1 CD004932 10.1002/14651858.CD004932.pub2 PMC12042072 17253532

[B14] Treating Opportunistic Infections Among HIV-Infected Adults and Adolescents http://www.cdc.gov/mmwr/preview/mmwrhtml/rr5315a1.htm

[B15] Carr A Marriott D Field A Vasak E Cooper DA Treatment of HIV-1-associated microsporidiosis and cryptosporidiosis with combination antiretroviral therapy Lancet 1998 351 256 61 10.1016/S0140-6736(97)07529-6 9457096

[B16] Maggi P Larocca AM Quarto M Serio G Brandonisio O Angarano G Pastore G Effect of antiretroviral therapy on cryptosporidiosis and microsporidiosis in patients infected with human immunodeficiency virus type 1 Eur J Clin Microbiol Infect Dis 2000 19 213 17 10.1007/s100960050461 10795595

